# From Intelligent Operating Room to Smart ICU: Digital Continuity, AI-Supported Decision-Making and CRRT as a Model of Pharmacokinetic Personalization in Critical Care

**DOI:** 10.3390/healthcare14172834

**Published:** 2026-09-03

**Authors:** Leonard Azamfirei, Mirela Cecilia Oiaga, Mihai Claudiu Pui

**Affiliations:** 1 Department of Anesthesiology and Intensive Care, George Emil Palade University of Medicine, Pharmacy, Science, and Technology of Targu Mures, 540142 Targu Mures, Romania; leonard.azamfirei@umfst.ro; 2 Doctoral School of Medicine and Pharmacy, George Emil Palade University of Medicine, Pharmacy, Science and Technology of Targu Mures, 540139 Targu Mures, Romania; morar.mirela-cecilia.22@stud.umfst.ro; 3 Department of Anesthesiology and Intensive Care Medicine, Emergency County Hospital, 540136 Targu Mures, Romania

**Keywords:** perioperative critical care, digital continuity, artificial intelligence, continuous renal replacement therapy, digital twin

## Abstract

Critically ill patients frequently move between the intensive care unit (ICU) and the operating room while remaining dependent on ventilatory, hemodynamic, antimicrobial, sedative, analgesic, and renal support. Although extensive perioperative data are generated, clinically relevant information may remain fragmented across devices and electronic systems. This narrative review synthesizes the literature published primarily between 2016 and 2026 on perioperative digital continuity, structured ICU–operating room handoff, interoperability, artificial intelligence (AI)-supported decision support, continuous renal replacement therapy (CRRT)-related pharmacokinetic personalization, and digital twin concepts. Based on this synthesis, we develop a conceptual framework in which the Intelligent Operating Room and Smart ICU function as connected clinical information nodes. The framework is organized around a minimum perioperative continuity dataset, a perioperative delta view, and the author-proposed concept of Time-to-Truth at ICU readmission. Current evidence supports structured handoff and selected AI-assisted physiological prediction, but evidence for improvement in major patient-centered outcomes remains limited and heterogeneous. CRRT illustrates the importance of preserving information on delivered therapy, interruptions, residual kidney function, and treatment-related changes when interpreting drug exposure. The proposed framework is not clinically validated and is intended as a basis for future implementation and prospective evaluation. Overall, the review suggests that improving digital continuity should precede more complex forms of automation in perioperative critical care.

## 1. Introduction

In Intensive Care, the clinical value of artificial intelligence depends closely on the quality and context of the data used. Predictive algorithms have value only if they accurately reflect the patient’s physiological evolution and can be effectively integrated into the medical decision-making process. This is very important in perioperative intensive care, where the same critically ill patient may leave the ICU for an operation and return a few hours later with altered parameters, different ventilatory needs, altered fluid balance, exposure to antimicrobials, analgesia, sedation, or renal support. For the patient, all these changes are part of a single physiological evolution. In hospital information systems, however, data are often divided among ICU documentation, anesthesia records, surgical notes, laboratory platforms, infusion pumps, ventilators, and renal replacement therapy devices [1,2,3].

This fragmentation becomes important during the ICU–operating room–ICU transfer. For the critically ill patient, the operating room is not just a separate procedural space, but a stage of the same critical illness. Intraoperative hypotension, bleeding, transfusion, changes in vasoactive support, ventilation strategy, timing of antibiotic administration, fluid therapy, and source control can influence postoperative treatment in the ICU. However, when the patient returns to the ICU, these data are not always immediately available in a structured, synchronized, and clinically easy-to-use form. The transfer of the patient from the operating room to the ICU is considered a vulnerable transition, and structured methods have been shown to improve the completeness and safety of information transfer [4,5]. This fragmentation creates a delay between data generation and clinically usable understanding [5]. The concept of the Intelligent Operating Room must be viewed in this context. Its value does not lie only in modern equipment, digital displays, video integration, or automatic data collection. For the critically ill patient, an operating room is truly intelligent when it helps maintain continuity of care by preserving the intraoperative physiological and therapeutic trajectory and making it available to the ICU team after transfer [6]. The same principle applies to the Smart ICU. An ICU with a large amount of data is not inherently intelligent for clinical decision-making. Data integration and visualization technologies can help clinicians by reducing fragmentation, cognitive burden, and the need to manually reconstruct the patient’s condition across different sources [7].

Artificial intelligence can help move from reactive monitoring to anticipatory care, but only if it is integrated into reliable clinical data flows. Machine-learning-based early warning systems for intraoperative hypotension show this potential, demonstrating that high-frequency physiological signals can be used to anticipate hemodynamic deterioration before conventional alarms are activated [8]. An example of clinical complexity in the ICU is the need for personalization in critically ill patients receiving CRRT, where drug exposure depends on patient factors, drug properties, and extracorporeal clearance, including modality, membrane characteristics, flow rates, filter lifespan, and the difference between the prescribed and delivered dose. These variables make standard dosing assumptions unsafe and support the use of therapeutic drug monitoring, pharmacokinetic modeling, and individualized dosing strategies, especially for drugs with a narrow therapeutic index or significant clinical risks associated with underexposure or accumulation [9]. Digital twin concepts could, in the future, connect these aspects by integrating physiology, treatment exposure, extracorporeal therapies, and predicted response into dynamic models specific to each patient. These systems are still largely under development in intensive care. For now, their relevance is mainly conceptual: they suggest a future in which the Intelligent Operating Room, Smart ICU, structured transfer, artificial-intelligence-assisted prediction, and pharmacokinetic personalization are part of the same continuity-based model of care [10].

Although digitalization is advancing in both the operating room and the intensive care unit (ICU), these environments are often developed and evaluated as distinct information ecosystems [6,7]. As a result, the increasing availability of perioperative data does not guarantee the continuity of clinically relevant information during transitions of critically ill patients between these settings [3,5,7]. This disconnect between digital data availability and clinically actionable continuity underpins the rationale for this review and the proposed framework focused on the ICU–operating room–ICU pathway.

The objective of this narrative review is to synthesize current evidence on digital continuity across the ICU–operating room–ICU pathway and, based on this synthesis, to develop a conceptual framework for smart perioperative critical care. The proposed framework does not represent a clinically validated model; rather, it integrates existing evidence on structured handoff, interoperability, AI-supported decision support, and pharmacokinetic personalization into a continuity-based approach centered on the critically ill patient. Its principal contribution is the organization of these previously separate domains around a minimum perioperative continuity dataset, a perioperative delta view, and the reduction in Time-to-Truth at ICU readmission. Within this framework, the Intelligent Operating Room and Smart ICU are considered connected clinical information nodes, AI-supported prediction is positioned as a decision-support layer, CRRT is used as a clinical example of pharmacokinetic personalization, and digital twin concepts are discussed as a future research direction.

## 2. Literature Search and Narrative Synthesis

This article was designed as a narrative review and conceptual framework, based on a thematic search of the literature, focusing on publications relevant to the digital continuity of care for the critically ill patient along the ICU–operating room–ICU pathway. The literature search focused on publications from 2016 to 2026. A final literature update was conducted on 31 August 2026. This period was chosen to reflect recent advances in perioperative digitalization, interoperability, artificial intelligence, and data-driven critical care. Earlier seminal publications were considered when necessary to support foundational concepts.

The original literature identification was conducted iteratively using PubMed as the primary bibliographic database, complemented by Google Scholar and reference-list screening. Google Scholar was used to identify additional conceptual articles, recent reviews, and other relevant publications not retrieved initially through PubMed. The search covered the following thematic domains: intelligent and digital operating rooms; OR–ICU handoff and perioperative continuity; interoperability and electronic health records; artificial intelligence and machine learning in anesthesia and critical care; intraoperative hypotension prediction; CRRT-related pharmacokinetics and drug dosing; model-informed precision dosing, therapeutic drug monitoring, and Bayesian dosing; digital twin concepts; emerging large language model and agentic AI applications; and healthcare interoperability standards. Because the initial search was conducted iteratively for a narrative review, the complete original query history was not prospectively retained. To improve transparency and provide a reproducible search component during revision, the final literature update used structured PubMed search strategies. The complete search strategies, including Boolean operators, field restrictions, language restriction, and publication-date limits, are provided in Supplementary Table S1.

Articles were included if they addressed perioperative or critical care settings relevant to the ICU–operating room–ICU pathway and examined at least one of the following domains: digital health technologies, interoperability, clinical decision support, artificial intelligence, continuous renal replacement therapy, pharmacokinetic personalization, or digital twin concepts. Eligible publications included original clinical studies, methodological or technological studies, consensus or guideline-based publications, and relevant conceptual or review articles. Only English-language publications were considered. Articles were excluded if they were not relevant to perioperative or critical care continuity, addressed technical or engineering aspects without clear clinical relevance to the proposed framework, provided insufficient information for the narrative synthesis, or represented duplicate publications.

Duplicate records identified across the searched sources were removed during reference screening and before the final thematic synthesis. Because this was a narrative rather than a systematic review, a separate numerical count of duplicate publications was not maintained. A single author (M.C.P.) conducted the screening process using a thematic relevance-based approach.

Potentially relevant publications identified through PubMed, complementary Google Scholar searches, and reference-list screening were assessed against the inclusion and exclusion criteria. Titles and abstracts were screened initially, followed by full-text assessment when eligibility or thematic relevance could not be determined from the abstract alone. Because the original literature identification was conducted iteratively for a narrative review, source-specific retrieval and exclusion counts were not prospectively recorded, and a systematic-review-style numerical flow was therefore not reconstructed retrospectively. Publications meeting the eligibility criteria and contributing directly to at least one of the thematic domains were retained for the narrative synthesis. To distinguish primary from secondary evidence more clearly, selected original research studies are summarized in Table 1, while representative review articles are presented separately in Table 2. Inclusion in either table was intended solely to provide a structured overview of the literature and did not constitute an additional eligibility or screening step.

The strength of the available evidence differed across the thematic domains. Randomized trials and systematic reviews were available in some areas, particularly AI-supported hemodynamic prediction and model-informed precision dosing, whereas other topics were supported mainly by observational, implementation, methodological, review, or conceptual studies. These differences in study design and level of validation were considered when interpreting the literature. No formal quality assessment or risk-of-bias evaluation was performed. This decision reflected the narrative design of the review and the heterogeneity of the included publication types. Instead, study design and level of validation were considered qualitatively when interpreting the evidence.

Artificial intelligence assistance was used during manuscript preparation for structural refinement, editorial suggestions, and assistance in preparing conceptual figure drafts. All AI-assisted outputs were critically reviewed, verified, and edited by the authors.

As shown in Table 2, the representative reviews selected for comparison generally address these domains separately. The present review brings these areas together within a continuity-based ICU–operating room–ICU framework, while recognizing differences in the supporting evidence and level of clinical validation across domains.

## 3. The ICU–OR–ICU Pathway: One Patient, One Physiology, Fragmented Data

For critically ill patients, surgery is part of a continuous process of organ dysfunction, therapeutic support, and clinical risk. Patients may arrive in the operating room already mechanically ventilated, on vasopressors, antimicrobials, sedation, or renal replacement therapy. Intraoperatively, their condition can change suddenly due to blood loss, fluid shifts, transfusion, ventilation adjustments, vasoactive support, or procedural complications. On return to the ICU, postoperative management is based on both clinical assessment and understanding intraoperative changes. Clinical systems often fragment this continuous trajectory into disparate information. ICU notes, anesthesia records, surgical documentation, laboratory results, and device data are not found in a unified picture. Some data is collected automatically, some manually or verbally, and some remains only on devices. Thus, the ICU team must reconstruct the perioperative pathway from multiple sources, often under time pressure and while managing patient instability. This process directly influences therapy decisions.

Patient handoff between the operating room and the ICU is essential for patient safety. Studies show that transitions are vulnerable to information loss and that structured handoffs improve communication [4,5]. The human-factors-centered approach reveals the importance of team roles, interruption management, and the order of steps [13]. Checklists and structured protocols have led to more complete information transfers and more organized teams, especially in cardiac surgery and pediatric ICU [14,15]. The quality of the handoff depends on the information transmitted and the system in which it occurs. However, standardization is not effective without digital integration. Protocols can fail when they rely only on memory or manual transcription. Implementation depends on the local context, workload, and team behavior [16]. Structured and sustainable tools are needed to maintain adherence [27]. Risk analyses show that handoff between the operating room and the ICU is a system problem, not only an issue of individual communication [28].

**Figure 1:**
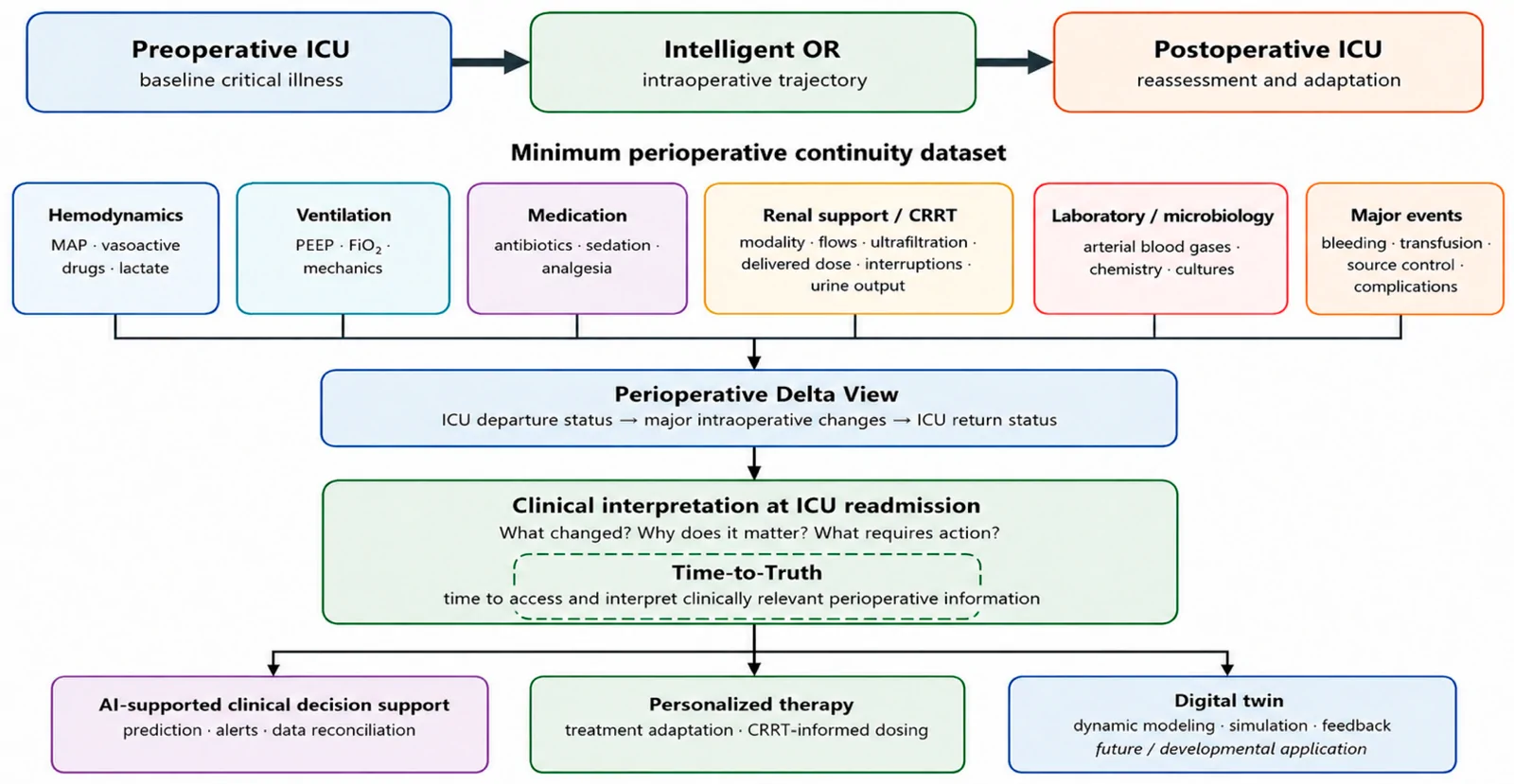
Proposed continuity-based framework for smart perioperative critical care across the ICU–operating room–ICU pathway. Clinical information is captured throughout the perioperative trajectory and organized within a minimum perioperative continuity dataset. At ICU readmission, these data support a perioperative delta view comparing ICU departure status, major intraoperative changes, and ICU return status. This change-focused representation is intended to facilitate clinical interpretation and provides the basis for the author-proposed concept of Time-to-Truth. AI-supported clinical decision support, personalized therapy, and digital twin applications are shown as downstream uses of the same structured information architecture rather than as sequential stages of care. Arrows and connecting lines indicate the direction and continuity of information flow, while colors are used to visually distinguish the different components of the framework. AI, artificial intelligence; CRRT, continuous renal replacement therapy; FiO_2_, fraction of inspired oxygen; ICU, intensive care unit; MAP, mean arterial pressure; OR, operating room; PEEP, positive end-expiratory pressure.

Time-to-Truth is defined as the time required for the receiving ICU team to understand what changed during the perioperative interval, why it changed, and how these changes should influence immediate treatment. In this framework, Time-to-Truth is an author-proposed, non-validated concept. For future validation, it could be operationalized as the interval between ICU readmission and the point at which the receiving team has access to, and can interpret, the key perioperative information required for immediate clinical decisions. It is not simply a measure of whether information was recorded, but of whether it becomes available, structured, and clinically interpretable at the moment of decision-making. In practical terms, prolonged Time-to-Truth may result from missing timestamps, incomplete documentation, inaccessible device data, or loss of contextual information during handover. In critically ill patients with shock, respiratory failure, sepsis, or continuous renal replacement therapy (CRRT), such delays may affect clinical decision-making regarding vasopressors, ventilation, antimicrobial therapy, sedation, analgesia, and renal support [27,28]. The proposed continuity-based model is summarized in Figure 1.

## 4. Intelligent Operating Room and Smart ICU as Connected Clinical Intelligence Nodes

The Intelligent Operating Room and the Smart ICU should be regarded not as distinct technological environments, but as interconnected clinical information nodes within a unified perioperative pathway. Their value derives primarily from their capacity to preserve, contextualize, and transmit clinically relevant information throughout the ICU–operating room–ICU transition, rather than from the isolated presence of advanced devices. For critically ill patients, the operating room represents a continuation of the existing physiological trajectory rather than an isolated episode. Intraoperative events such as hypotension, bleeding, transfusion, changes in ventilation, vasoactive support, antimicrobial timing, sedation, analgesia, or renal replacement therapy can directly influence subsequent ICU management decisions. The primary function of the Intelligent Operating Room is to capture these events, temporally align them, and convey their clinical significance to the ICU team [6,11,12,29]. The Smart ICU serves a complementary role by integrating intraoperative information with the patient’s preoperative status and postoperative progression. An increased volume of data does not inherently enhance decision-making if the information remains fragmented, poorly synchronized, or difficult to interpret. Data integration and visualization tools are effective when they reduce cognitive burden and enable clinicians to rapidly comprehend the patient’s clinical trajectory [7,17,30].

Within this model, the Intelligent Operating Room and Smart ICU are linked through a shared information architecture. The primary objective extends beyond digital documentation to encompass continuity of clinical reasoning. One practical manifestation of this connection is the perioperative delta view, which summarizes clinically relevant changes occurring between ICU departure, the intraoperative period, and ICU return. This approach is intended to facilitate more rapid recognition of significant changes and support more coherent postoperative decision-making, drawing on principles established in structured handoff literature [4,5,13,14,15,16,27,28].

The Time-to-Truth and perioperative delta view presented in this framework are conceptual constructs developed from the narrative synthesis. These constructs have not yet been subjected to pilot implementation, case-based evaluation, or formal expert validation. Potential validation measures could include time to retrieval of essential perioperative information, completeness of the continuity dataset, time to treatment-related decisions, and clinician agreement on the relevance of the information provided. In contrast, the Intelligent OR and Smart ICU concepts discussed in this review are grounded in the existing literature, including conceptual, implementation, and clinical studies.

### 4.1. Perioperative Delta View

The perioperative delta view is a proposed practical tool designed to shorten Time-to-Truth. It does not replace the full anesthesia record or ICU documentation; instead, it summarizes clinically relevant differences among ICU departure, the intraoperative period, and ICU return. This summary enables the receiving ICU team to quickly identify changes that require therapeutic action (Table 3).

### 4.2. Minimum Perioperative Continuity Dataset

For an effective perioperative pathway, it is important to define and maintain a focused set of essential continuity data. This information should be consistent, easy to understand in a clinical setting, and not rely only on verbal handoffs or scattered electronic records [3,7]. This set should include variables relevant for immediate postoperative management: the patient’s condition before transfer, hemodynamic evolution, ventilation strategy, medication and infusion changes, renal and CRRT data, laboratory and microbiology data, important intraoperative events, and unresolved clinical problems. Studies show that structured approaches can reduce information loss during transfer and improve the completeness and organization of handoff communication [5,6,13,14,15,16,27,28].

Hemodynamic data, such as trends in mean arterial pressure, vasopressor doses, fluids, blood loss, and lactate dynamics, provide vital context compared with a single static postoperative value. Similarly, ventilator data must include relevant changes in oxygenation, PEEP, driving pressure, and episodes of hypoxemia, influencing decisions regarding extubation and lung protection [7,8]. Continuity of medication information is critical: the timing of antimicrobial administration, perioperative exposure to analgesics, sedatives, or anticoagulants, and infusion changes can directly influence recovery and the risk of complications, especially in critically ill patients [3,5,9]. For renal function and CRRT, it is essential that details such as the type of therapy, flow rates, ultrafiltration, filter age, and circuit interruptions are transferred, since these influence drug dosing and clearance [9,30].

**Figure 2:**
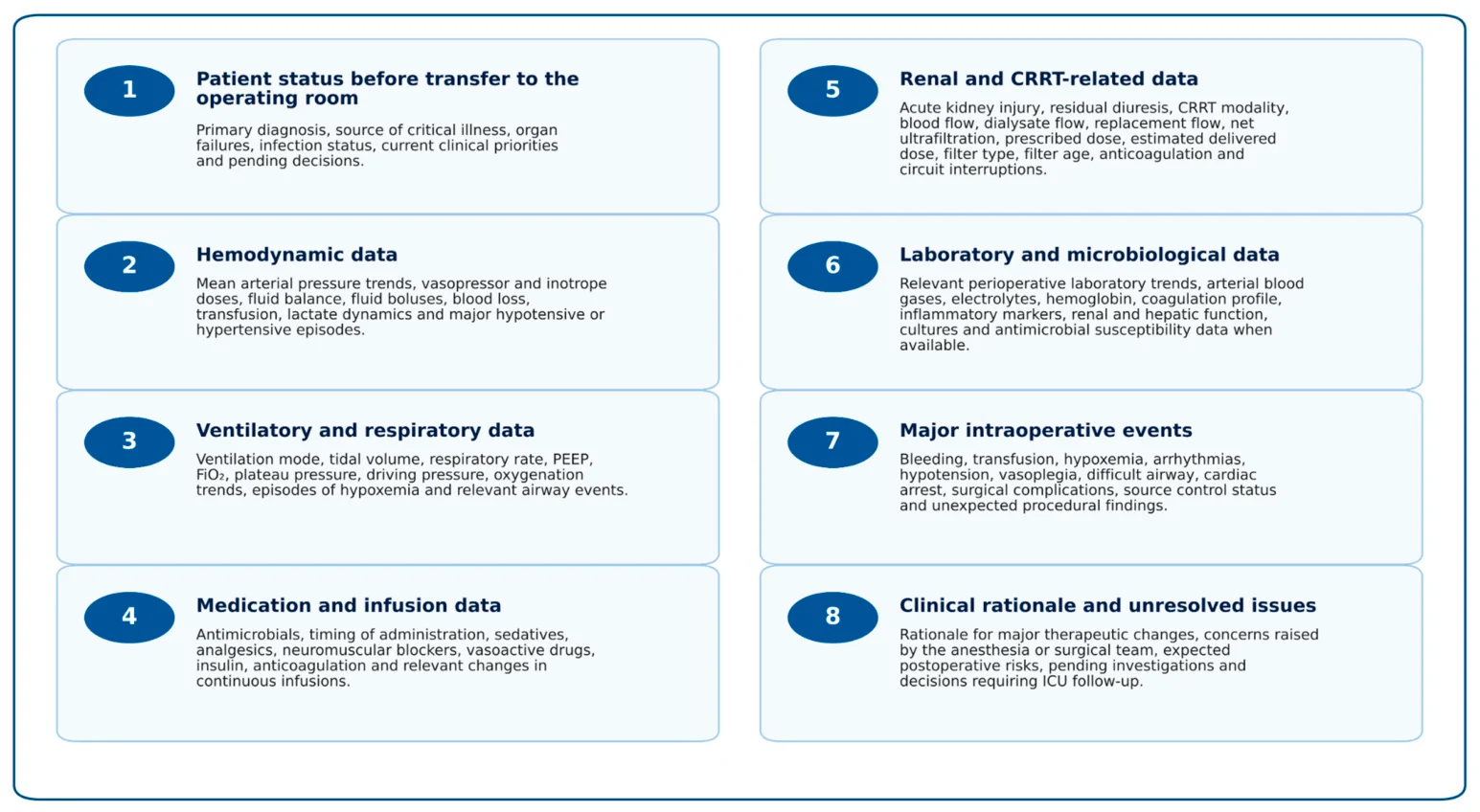
Minimum ICU-OR-ICU continuity dataset. CRRT, continuous renal replacement therapy; FiO_2_, fraction of inspired oxygen; ICU, intensive care unit; OR, operating room; PEEP, positive end-expiratory pressure.

Finally, clinical reasoning must accompany the numerical data: the justification of major interventions and the mention of unresolved problems or persistent risks are crucial for safe postoperative decisions [5,6,13,16,27,28]. The minimum perioperative continuity dataset serves as the data foundation for generating the perioperative delta view. Its purpose is not to duplicate the handover summary, but to define the essential variables that must be continuously captured, updated, and accessible throughout the ICU–OR–ICU pathway [3,5,7,13,16]. The delta view then uses these variables to identify clinically relevant changes and immediate therapeutic implications upon ICU return [4,5,13,14,15,16,27,28] (Figure 2). The proposed framework is designed to complement, rather than replace, existing structured handoff protocols and interoperability models. Structured handoffs standardize the information communicated during care transitions, whereas interoperability facilitates data exchange between clinical systems. The minimum perioperative continuity dataset and delta view introduce a longitudinal, change-focused dimension by emphasizing clinically significant differences between preoperative and postoperative states. The primary contribution is not the creation of a new communication protocol or data standard, but rather the development of a clinically oriented approach to organizing existing information to reduce Time-to-Truth at ICU readmission.

### 4.3. Interoperability, EHR Integration and Clinical Decision Support

The continuity dataset becomes truly valuable only if it is accessible both technically and clinically. In many hospitals, relevant data are scattered across monitors, ventilators, pumps, electronic records, and other platforms, lacking interoperability and creating gaps between data generation and their practical use for critically ill patients transitioning between the ICU and the operating room [3,7]. Interoperability is not only a technological objective, but an important condition for safe and effective information exchange across care settings. Without rapid access to and direct correlation of perioperative data, clinical teams end up manually reconstructing the patient’s history under emergency conditions, with an increased risk of errors, especially when essential variables such as vasopressors, fluids, antimicrobials, or ventilation change rapidly. Studies in intensive care emphasize the importance of data harmonization and governance, interoperability, and the ethical use of clinical information [3].

Effective implementation of the proposed framework necessitates alignment with established healthcare interoperability standards. HL7 FHIR facilitates structured exchange of clinical information, while standardized terminologies such as SNOMED CT and LOINC enhance semantic consistency across systems [31,32]. Integration with existing hospital information systems, robust cybersecurity measures, and clearly defined data governance and access control policies are also essential [3,31]. Integration of the electronic health record should help; however, in the absence of a relevant visual presentation, large volumes of perioperative and ICU data can become difficult to interpret. Analytical visualization, such as dashboards generated from anesthesia data, may support clinical decisions, performance monitoring, and feedback [17]. For the development of relevant artificial intelligence in the perioperative setting, detailed, structured, and temporally correlated datasets are necessary. Public databases such as MOVER demonstrate that electronic data can be correlated with physiological waveforms and high-fidelity clinical events [18]. Without this structure, AI will replicate the limitations of the fragmented system in which it was trained.

Along the ICU–operating room–ICU pathway, these data are vital for postoperative assessment of hemodynamic status, level of consciousness, and drug exposure. Successful implementation of perioperative digital systems requires real clinical involvement, not only IT or software input. The anesthesiologist–informatician may play an important role in this transformation, bridging anesthesia information systems, clinical decision support, and quality-improvement processes [33]. Device integration is particularly important for medication safety: communication between smart pumps and electronic records reduces administration errors and clarifies dose evolution for vasopressors, sedatives, insulin, or anticoagulants [34]. In an ideal model, informatics leadership must involve all professions: anesthesia, intensive care, surgery, pharmacy, nurses, and IT. Intelligent clinical decision support should be introduced only after these foundations are solid. If the data are unreliable or the workflow is not intuitive, alert systems can increase team stress without providing real benefits [2]. Only after interoperability, structure, and clinical visualization are in place can AI and decision support deliver value. In this context, the Intelligent Operating Room and Smart ICU are connected through a shared information architecture designed to reduce fragmentation, shorten Time-to-Truth, and support safer perioperative decision-making [2].

## 5. AI-Supported Hemodynamic Prediction: From Reactive Monitoring to Anticipatory Care

Hemodynamic instability continues to pose a significant perioperative risk for critically ill patients, particularly during transitions between the intensive care unit and the operating room. Intraoperative hypotension can arise from factors such as hypovolemia, vasodilation, bleeding, cardiac dysfunction, anesthetic depth, inflammation, or ventilatory changes. While conventional monitoring typically provides reactive responses, contemporary hemodynamic monitoring aims to facilitate earlier detection of deterioration and guide interventions to maintain organ perfusion [35]. Machine learning-based systems designed to predict intraoperative hypotension demonstrate the potential of artificial intelligence to enhance anticipatory care [36,37]. The HYPE randomized clinical trial found that an early warning system utilizing machine learning, in conjunction with a therapeutic protocol, reduced both the duration and severity of intraoperative hypotension compared to standard care [8]. Subsequent reviews indicate that AI-supported perioperative interventions may improve certain physiological endpoints, particularly the burden of hypotension; however, evidence regarding patient-centered outcomes such as organ injury, ICU length of stay, or mortality remains limited [24,38,39,40,41].

A primary limitation is that prediction alone is not sufficient. Alerts indicating potential hypotension offer limited clinical value unless they are contextualized within the patient’s physiological status and linked to actionable therapeutic strategies. Similar decreases in arterial pressure may result from hypovolemia, vasodilation, myocardial dysfunction, bleeding, or excessive anesthetic depth, each necessitating a distinct intervention. Consequently, AI tools should be interpretable, clinically integrated, and embedded within the clinical workflow rather than operating as isolated alert systems. Clinical implementation further requires representative data, external validation, assessment of uncertainty and bias, human oversight, and compliance with evolving regulatory and data-governance requirements [42,43].

Within the proposed framework, the evaluation of AI-supported hemodynamic prediction should consider not only its capacity to anticipate hypotension but also whether it helps clinicians recognize and interpret relevant physiological changes more rapidly [19,38,39,44,45]. Large language models and other generative AI tools may support perioperative continuity by reconciling information from clinical notes, medications, laboratory results, and device data into concise summaries at transitions of care [25,46]. Agentic AI could extend this role by identifying missing information, tracking treatment changes, and supporting context-aware retrieval across the ICU–operating room–ICU pathway [47]. However, these applications remain largely exploratory and require human oversight, external validation, reliable interoperability, and appropriate governance before routine clinical use [25,42,43,47].

## 6. CRRT as a Model of Pharmacokinetic Complexity in Critical Care

Continuous renal replacement therapy (CRRT) provides a practical model for pharmacokinetic personalization in critically ill patients, as unstable physiology and extracorporeal clearance can lead to significant variability in drug exposure [9,48,49,50,51,52]. Drug clearance during CRRT depends on both the treatment modality and the intensity of delivered therapy. Continuous venovenous hemofiltration (CVVH) relies predominantly on convective clearance, continuous venovenous hemodialysis (CVVHD) on diffusive clearance, and continuous venovenous hemodiafiltration (CVVHDF) combines both mechanisms. For pharmacokinetic interpretation, relevant treatment data therefore include CRRT modality, blood flow rate, dialysate flow, replacement-fluid flow, pre- or post-dilution, and the delivered effluent dose, preferably expressed in mL/kg/h [30,48,49,50,51,52]. The prescribed CRRT dose should be distinguished from the dose actually delivered, as interruptions, circuit clotting, filter changes, and other periods of downtime may substantially reduce effective treatment time and extracorporeal clearance [30,48,49].

Drug exposure also depends on factors not captured by effluent dose alone. Residual kidney function, including urine output and, when available, an estimate of intrinsic renal clearance, contributes to total drug elimination. Membrane and circuit characteristics may further modify extracorporeal drug removal, including through adsorption, particularly for drugs with relevant protein binding or membrane interactions [48,49,50,51,52]. When therapeutic drug monitoring is available, the timing of sampling should be interpreted in relation to drug administration, CRRT settings, and recent interruptions or changes in treatment delivery [50,51]. Antimicrobial therapy exemplifies these challenges most clearly. Underdosing can result in therapeutic failure and promote antimicrobial resistance, while overdosing increases the risk of toxicity. Extracorporeal clearance is determined by drug-specific factors, including molecular weight, protein binding, and volume of distribution, as well as CRRT-specific factors such as modality, membrane type, and delivered dose [50,51,52]. Comparable considerations apply to sedatives and analgesics, where insufficient exposure may cause agitation, pain, and ventilator asynchrony, whereas excessive exposure can lead to prolonged sedation, respiratory depression, and delirium [26,53,54,55,56].

CRRT demonstrates the clinical importance of digital continuity in perioperative critical care. Accurate interpretation of drug exposure requires consideration of the patient’s physiological trajectory, CRRT prescription, delivered dose, treatment interruptions, circuit performance, and clinical response, rather than relying solely on the last administered dose or renal function [9,30,50,51,52]. Therefore, CRRT-related variables should be incorporated into the minimum perioperative continuity dataset and the perioperative delta view because they influence drug exposure and may require reassessment of antimicrobial, sedative, and analgesic dosing. For example, if CRRT is interrupted during surgery, documenting the duration of the interruption, circuit status, and treatment actually delivered allows the receiving team to reassess the likely change in extracorporeal drug clearance. Such documentation may prompt reassessment of drug exposure and subsequent dosing according to drug-specific pharmacokinetics, residual kidney function, the duration of CRRT interruption, and the availability of TDM, rather than relying on the assumption of uninterrupted CRRT based on the preoperative prescription [20,26,30,51,53,54,55,57,58]. Future work should include Delphi-based consensus studies to validate and prioritize practical dosing recommendations for drugs used during CRRT and other extracorporeal blood purification techniques.

### Model-Informed Precision Dosing, Therapeutic Drug Monitoring and Bayesian Dosing

Drug dosing in critically ill patients is influenced by rapidly changing physiology, organ dysfunction, inflammation, fluid shifts, and extracorporeal therapies. Therapeutic drug monitoring yields measured drug concentrations and can detect underexposure or accumulation; however, a concentration value alone does not necessarily indicate the optimal subsequent dose. This consideration is especially relevant for patients receiving continuous renal replacement therapy (CRRT), as renal function, extracorporeal clearance, and the delivered treatment dose may fluctuate over short intervals [21,59]. Bayesian dosing offers a systematic approach for interpreting measured drug concentrations in the context of individual patient characteristics. This method integrates population pharmacokinetic models with patient-specific data, dosing history, measured concentrations, and, when applicable, CRRT parameters. The objective is to estimate current drug exposure and predict the dose most likely to achieve a predefined pharmacokinetic/pharmacodynamic (PK/PD) target [22,60,61].

Model-informed precision dosing (MIPD) represents the broader clinical framework for applying this process. In practice, the workflow involves integrating clinical data, dosing history, CRRT parameters, and measured concentrations into a PK/PD model. The model estimates drug exposure, generates an individualized dose recommendation, and prompts reassessment following subsequent clinical or pharmacokinetic changes. The primary clinical justification for this approach is antimicrobial therapy, particularly for beta-lactams and other agents with well-defined PK/PD targets. Nevertheless, existing studies indicate that improved target attainment does not necessarily result in better patient-centered outcomes. Implementation is further constrained by delayed assays, uncertain targets, software usability challenges, workflow integration issues, and the need for local model validation [21,22,59,60,61]. A similar rationale may extend to sedatives, analgesics, and selected anesthetic agents, although the supporting evidence is less robust compared to antimicrobials. For these medications, pharmacokinetic models should be interpreted alongside clinical objectives, including pain control, depth of sedation, delirium prevention, respiratory recovery, and hemodynamic stability [26,53,54,55,62].

**Figure 3:**
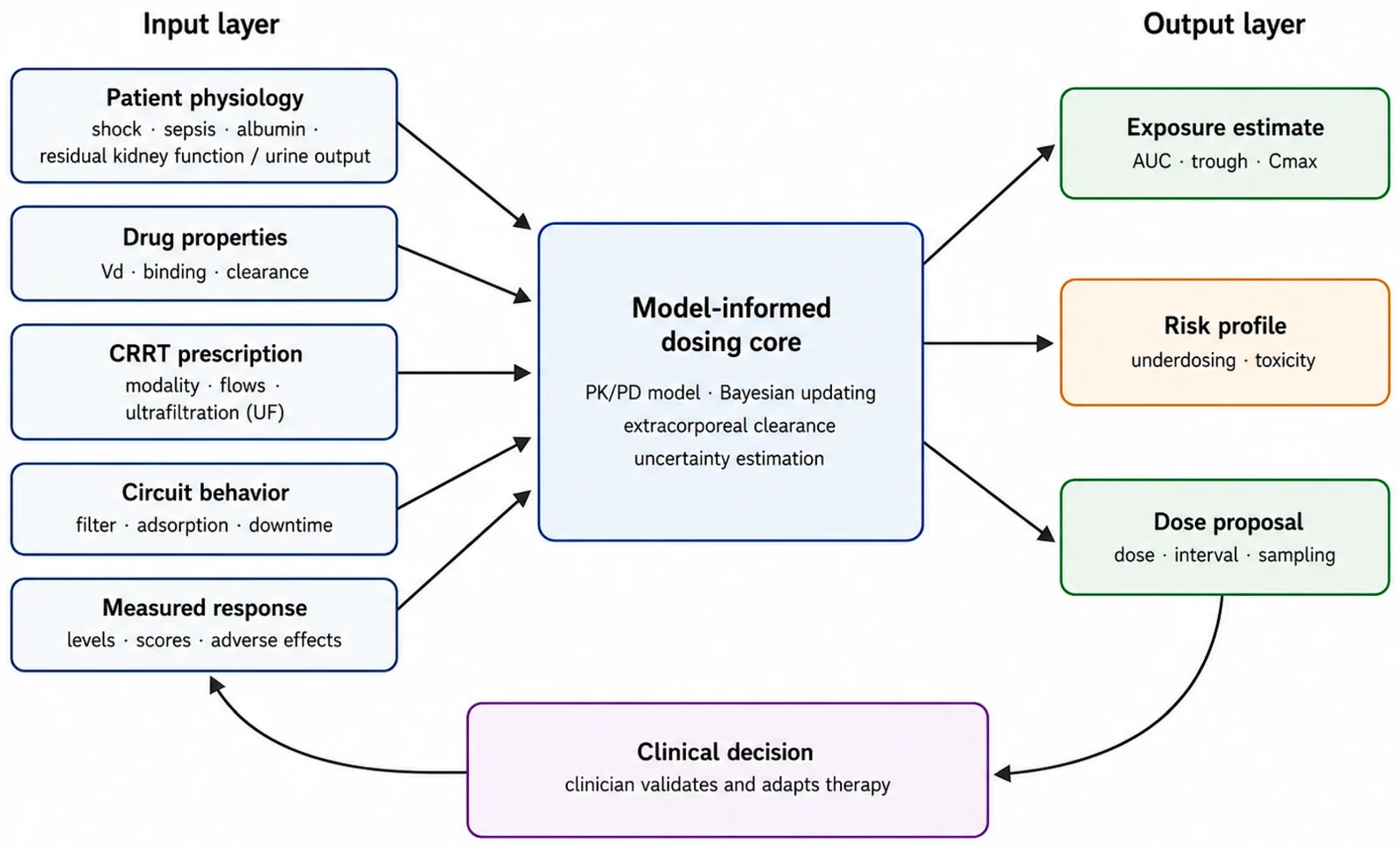
Proposed model-informed precision dosing workflow for individualized drug dosing during CRRT. AUC, area under the concentration–time curve; Cmax, maximum concentration; CRRT, continuous renal replacement therapy; PK/PD, pharmacokinetic/pharmacodynamic; UF, ultrafiltration; Vd, volume of distribution. Arrows indicate the direction of information flow and the iterative feedback process, while colors are used to distinguish the different functional components of the workflow.

Within the intensive care unit (ICU)–operating room–ICU pathway, MIPD is pertinent because drug exposure may fluctuate during surgery due to altered physiology, interrupted CRRT, transfusion, fluid administration, delayed antimicrobial dosing, or modifications in sedation and analgesia. Consequently, precision dosing should be conceptualized as a dynamic feedback process rather than a one-time dose adjustment. Its effectiveness relies on reliable clinical data, timely therapeutic drug monitoring (TDM), validated models, clear data visualization, and seamless integration into the clinical workflow [63,64,65] (Figure 3).

## 7. Digital Twin of the Critically Ill Patient: Physiological and Pharmacokinetic Layers

A digital twin should be distinguished from a conventional predictive model or a simulation tool. Predictive models estimate future outcomes from available data, while simulation tools explore hypothetical scenarios without necessarily maintaining a continuously updated representation of an individual patient. In contrast, a true digital twin requires repeated or continuous updating with patient-specific data so that the virtual representation evolves with the clinical state. In critical care, most current applications remain conceptual, developmental, or research-oriented rather than fully implemented bedside digital twins [10,23,66,67,68].

The development of a clinically meaningful digital twin requires the integration of heterogeneous, time-dependent data, including physiological signals, laboratory results, medication exposure, and organ-support settings. Once dynamically updated with patient-specific data, such models may support simulation of clinical trajectories and estimation of individualized responses to interventions. However, successful clinical implementation depends on reliable data integration, temporal synchronization, rigorous model validation, and effective management of predictive uncertainty [10,23,68,69]. In the ICU–operating room–ICU pathway, the digital twin concept is useful because it highlights the importance of continuity of care. A perioperative digital model should track the same patient throughout critical illness, surgery, and ICU readmission, integrating data on hemodynamics, ventilation, fluid balance, laboratory dynamics, organ support, antimicrobial exposure, sedation, analgesia, and continuous renal replacement therapy (CRRT). Here, surgery is considered an integral component of the overall physiological and therapeutic trajectory, rather than a discrete episode.

Two complementary layers are relevant to this approach. The physiological layer encompasses hemodynamics, respiratory status, organ dysfunction, inflammation, laboratory trends, and treatment response. The pharmacokinetic layer comprises administered doses, timing, CRRT parameters, treatment interruptions, measured drug concentrations, pharmacokinetic/pharmacodynamic (PK/PD) targets, and clinical response. This pharmacokinetic layer links therapeutic drug monitoring, Bayesian forecasting, and model-informed precision dosing. Current digital twin applications in critical care therefore remain primarily research-oriented rather than routine bedside decision-support tools [23,66,67,68,69,70].

## 8. Implementation Barriers, Governance and Roadmap

In intelligent perioperative intensive care, the main challenge is in integrating existing clinical data into a coherent system that supports bedside decision-making. Devices such as monitors, ventilators, infusion pumps, laboratory platforms, electronic records, and continuous renal replacement therapy (CRRT) systems already generate relevant information. However, these data are frequently incomplete, poorly synchronized, or unavailable at critical moments for clinicians [3,7,17,18]. Implementation should be guided by clinical needs rather than algorithmic capabilities. The initial priority is to define the essential variables necessary for safe transitions between the intensive care unit (ICU) and the operating room, and to standardize the collection, display, and transfer of these data. Advanced functions, including predictive analytics, model-informed dosing, or digital twin applications, should be introduced only after these foundational elements are established. This approach aligns with responsible artificial intelligence (AI) principles that emphasize data quality, interoperability, and clinician involvement [42,43]. Multiple barriers must be addressed to achieve effective integration. Technical barriers include heterogeneous devices, inconsistent terminology, and limited interoperability. Organizational barriers encompass unclear responsibilities, insufficient training, and resistance to workflow changes. Clinical barriers involve alert fatigue, limited trust in non-transparent models, and concerns regarding automation bias [22,38,42,43]. Consequently, governance should be multidisciplinary, engaging intensivists, anesthesiologists, surgeons, pharmacists, nurses, informaticians, biomedical engineers, data security specialists, and hospital leadership [3,33,34,43].

A practical roadmap should proceed incrementally, beginning with standardized perioperative data transfer, integration of multiple data sources, development of a perioperative delta view, and implementation of dashboards designed to reduce cognitive burden. Decision support should initially focus on low-risk functions, such as reminders or simple alerts, before advancing to predictive or prescriptive tools. Each stage requires prospective evaluation and post-implementation monitoring [2,3,7,17,24,34].

Within this framework, the immediate objective is not full automation, but rather the preservation of the perioperative physiological and therapeutic trajectory in a structured format that facilitates handover, rapid interpretation, and coherent ICU decision-making. Ultimately, artificial intelligence should be regarded as the outcome of a mature data ecosystem rather than as an immediate solution. A central contribution of this framework is the emphasis on preserving and interpreting the patient’s clinical trajectory across different care environments [3,43,68,70]. Accordingly, the practical value of the framework at this stage lies in providing a structured basis for implementation and prospective evaluation rather than in representing an already validated clinical intervention.

## 9. Limitations

This review presents several limitations. First, it was structured as a narrative review and conceptual framework rather than a systematic review or meta-analysis. Consequently, no formal PRISMA-based selection process, quantitative synthesis, or meta-analysis was conducted. Literature selection was thematic and intended to support the development of the proposed ICU–operating room–ICU continuity model. In addition, the iterative literature identification and single-author thematic screening may have introduced selection bias, and the structured final literature update was limited to PubMed.

Second, the proposed framework has not undergone clinical validation and has not yet been evaluated through pilot implementation, simulation, expert consensus, or usability testing. Future research should incorporate pilot implementation and usability testing, followed by prospective clinical validation. The minimum perioperative continuity dataset, the perioperative delta view, and the integration of CRRT-related pharmacokinetic data should be regarded as conceptual and practical proposals rather than established standards of care.

Third, several components discussed in this review, including AI-supported prediction, model-informed precision dosing, and digital twin applications, are at varying stages of development and implementation. While some tools have demonstrated benefits for selected physiological endpoints, evidence remains limited for patient-centered outcomes such as mortality, ICU length of stay, long-term recovery, or cost-effectiveness.

Finally, implementation depends heavily on local infrastructure, electronic health record interoperability, device integration, data governance, staff training, and clinical acceptance. Therefore, the proposed model may not be directly generalizable to all institutions and should be adapted to local clinical workflows and available digital resources. In addition, part of the evidence supporting structured perioperative handoff processes derives from cardiac surgical and pediatric populations, which may limit direct extrapolation to heterogeneous adult surgical ICU populations.

Future clinical studies should evaluate the proposed framework through pilot implementation and prospective validation across the ICU–operating room–ICU pathway. Relevant outcomes for assessment include feasibility, data completeness, Time-to-Truth, handoff quality, clinician workload, and selected patient-centered outcomes. Subsequent multicenter studies may determine whether continuity-based digital integration enhances clinical decision-making, workflow efficiency, and clinical outcomes compared with current practice.

## 10. Conclusions

Smart perioperative critical care should be seen mainly as a problem of continuity, not only as a technological advance. A minimum continuity dataset and a perioperative delta view may help the ICU team understand what changed during surgery and what needs to be addressed immediately after return. AI-supported prediction, CRRT-based pharmacokinetic personalization and digital twin concepts may be useful only if they are built on reliable, structured and clinically relevant data. For now, the priority is not complete automation, but better preservation and interpretation of the patient’s perioperative trajectory. The proposed framework remains conceptual and requires prospective validation before its clinical impact can be established.

## Figures and Tables

**Table 1:** Selected original research studies included in the narrative synthesis, organized by thematic domain.

Domain	Reference	Article Type	Key Point
Intelligent OR/Digital OR	Carstensen et al., 2020 [11]	Implementation study	Integrated OR workflow.
Intelligent OR/Digital OR	Lin et al., 2020 [12]	Technical study	Real-time intraoperative data streaming.
OR–ICU handover	Dusse et al., 2021 [5]	Observational study	Variable handover completeness.
OR–ICU handover	Segall et al., 2016 [13]	Human-centered design study	Multidisciplinary handover redesign.
OR–ICU handover	Gleicher et al., 2017 [14]	Quality improvement study	Standardized cardiac OR–ICU handover.
OR–ICU handover	Malenka et al., 2018 [15]	Before–after QI study	Improved information transfer.
OR–ICU handover	Lane-Fall et al., 2023 [16]	Implementation study	Protocol fidelity determinants.
Interoperability/EHR	Kahn et al., 2022 [17]	Implementation/visual analytics study	Anesthesia EHR visual analytics.
Interoperability/EHR	Samad et al., 2023 [18]	Database resource	High-resolution OR database.
Hemodynamic prediction	Wijnberge et al., 2020 [8]	Randomized clinical trial	ML warning reduced hypotension burden.
Hemodynamic prediction	Kouz et al., 2023 [19]	ML analysis	Hemodynamic endotypes of IOH.
CRRT/drug dosing	Chen and Murugan, 2021 [20]	Survey study	Variability in CRRT ultrafiltration.
MIPD/precision dosing	Ewoldt et al., 2022 [21]	Multicentre RCT	MIPD did not improve major outcomes.
MIPD/precision dosing	Chai et al., 2024 [22]	Mixed-method study	MIPD usability barriers.
Digital twin	Lal et al., 2020 [23]	Development study	Sepsis digital twin model.

CRRT, continuous renal replacement therapy; EHR, electronic health record; ICU, intensive care unit; IOH, intraoperative hypotension; MIPD, model-informed precision dosing; ML, machine learning; OR, operating room; RCT, randomized controlled trial.

**Table 2:** Representative review articles relevant to the proposed continuity framework.

Review	Main Area	Main Contribution	Relation to the Present Review
Wheeler et al., 2018 [4]	OR–ICU handoff	Review of risks and approaches to improving transitions between the OR and ICU	Supports the handoff component; does not address the broader integration of perioperative digital continuity, AI, and pharmacokinetic personalization
Laterza et al., 2024 [6]	Smart operating room	Narrative review of Smart OR concepts and technologies	Provides the technological context for the Intelligent OR; the present review extends the focus to continuity with the ICU
Lin et al., 2019 [7]	ICU data integration	Systematic review and meta-analysis of data-integration technologies and clinician performance	Supports the role of integrated clinical information; does not specifically address the ICU–OR–ICU pathway
Mehta et al., 2024 [24]	Perioperative AI	Systematic review and meta-analysis of machine-learning-augmented perioperative interventions	Supports the AI decision-support component; the present review places these tools within a broader continuity framework
Shi et al., 2025 [25]	LLMs in critical care	Scoping review of large language model applications in critical care	Provides evidence for emerging AI-supported information processing; these applications represent only one component of the present framework
Jang et al., 2020 [9]	CRRT drug dosing	Review of drug-dosing considerations during CRRT	Supports the pharmacokinetic personalization component; the present review links CRRT-related information to perioperative digital continuity
Di Mario et al., 2025 [26]	Analgesic/sedative dosing during renal replacement therapy	Narrative review of analgesic and sedative dosing across renal replacement modalities	Supports the drug-exposure component of the framework, particularly for sedatives and analgesics
Zhang et al., 2024 [10]	Digital twin	Review of concepts and applications of digital twins in healthcare	Provides the conceptual basis for the digital twin section; the present review restricts this concept to its potential role within ICU–OR–ICU continuity

AI, artificial intelligence; CRRT, continuous renal replacement therapy; ICU, intensive care unit; LLM, large language model; OR, operating room.

**Table 3:** Proposed perioperative delta view for ICU–operating room–ICU continuity.

Component	ICU Departure Status	Major Intraoperative Changes	ICU Return Status	Immediate Therapeutic Implication
Hemodynamics	MAP, vasopressor dose, lactate	hypotension, vasopressor escalation, arrhythmias	current MAP, vasopressor requirement, lactate trend	fluid, vasopressor or inotrope adjustment
Ventilation	mode, FiO_2_, PEEP, mechanics	hypoxemia, airway event, ventilation change	current settings, gas exchange, respiratory mechanics	lung-protective strategy, extubation planning
Fluids and transfusion	fluid balance, hemoglobin, coagulation	blood loss, crystalloids, blood products	cumulative balance, hemoglobin, coagulation profile	bleeding risk, resuscitation strategy
Medication	antibiotics, sedation, analgesia, anticoagulation	delayed dose, redosing, infusion changes	current exposure and active infusions	antimicrobial reassessment, sedation and analgesia plan
CRRT	modality, flows, filter age, anticoagulation	interruption, filter change, circuit clotting, downtime	delivered dose, current flows, restart status	reassessment of drug exposure and dosing strategy, renal support plan
Surgical events	planned procedure and source control objective	complications, incomplete source control, reintervention risk	unresolved surgical concerns	monitoring priorities, need for reoperation or imaging
Laboratory/microbiology	lactate, pH, electrolytes, hemoglobin, cultures	new samples, abnormal results, pending cultures	current results, pending microbiology	correction of metabolic disorders, antimicrobial reassessment

CRRT, continuous renal replacement therapy; FiO_2_, fraction of inspired oxygen; ICU, intensive care unit; MAP, mean arterial pressure; PEEP, positive end-expiratory pressure.

## Data Availability

No new data were created or analyzed in this study. Data sharing is not applicable to this article.

## Supplementary Materials

The following supporting information can be downloaded at https://www.mdpi.com/article/10.3390/healthcare14172834/s1, Table S1: PubMed search strategies used for the final literature update conducted on 31 August 2026.

## Abbreviations

The following abbreviations are used in this manuscript:

AI	Artificial intelligence
CDSS	Clinical decision support system
CRRT	Continuous renal replacement therapy
EHR	Electronic health record
ICU	Intensive care unit
IOH	Intraoperative hypotension
MAP	Mean arterial pressure
MIPD	Model-informed precision dosing
ML	Machine learning
MOVER	Medical Informatics Operating Room Vitals and Events Repository
OR	Operating room
PEEP	Positive end-expiratory pressure
PK	Pharmacokinetics
LLM	Large language model
PD	Pharmacodynamics
PK/PD	Pharmacokinetic/pharmacodynamic
PRISMA	Preferred Reporting Items for Systematic Reviews and Meta-Analyses
HL7	Health Level Seven
QI	Quality improvement
RCT	Randomized clinical trial
RRT	Renal replacement therapy
TDM	Therapeutic drug monitoring
FHIR	Fast Healthcare Interoperability Resources
LOINC	Logical Observation Identifiers Names and Codes
SNOMED CT	Systematized Nomenclature of Medicine Clinical Terms
