# Supplementary material for: From Intelligent Operating Room to Smart ICU: Digital Continuity, AI-Supported Decision-Making and CRRT as a Model of Pharmacokinetic Personalization in Critical Care
Source: Healthcare (Basel). 2026 Sep 3;14(17):2834. doi: 10.3390/healthcare14172834 (PMC13566315; doi:10.3390/healthcare14172834)
Supplement: Supplementary file 1 [file healthcare-14-02834-s001.zip › healthcare-4523492-supplementary.pdf]

Supplementary Table S1. PubMed search strategies used for the final literature update conducted on 31 August 2026.

No.	Thematic domain	Complete PubMed search string
1	**Intelligent / Digital Operating Room**	("intelligent operating room"[Title/Abstract] OR "smart operating room"[Title/Abstract] OR "digital operating room"[Title/Abstract] OR "integrated operating room"[Title/Abstract] OR "hybrid operating room"[Title/Abstract]) AND ("2016/01/01"[Date - Publication] : "2026/08/31"[Date - Publication]) AND english[Language]
2	**OR–ICU Handoff / Perioperative Continuity**	(("operating room"[Title/Abstract] OR "operating theatre"[Title/Abstract] OR perioperative[Title/Abstract]) AND ("intensive care unit"[Title/Abstract] OR ICU[Title/Abstract]) AND (handoff[Title/Abstract] OR handover[Title/Abstract] OR transfer[Title/Abstract] OR transition*[Title/Abstract] OR "continuity of care"[Title/Abstract])) AND ("2016/01/01"[Date - Publication] : "2026/08/31"[Date - Publication]) AND english[Language]
3	**Interoperability / Electronic Health Records / Clinical Decision Support**	(("electronic health record"[Title/Abstract] OR "electronic health records"[Title/Abstract] OR EHR[Title/Abstract] OR interoperability[Title/Abstract] OR "data integration"[Title/Abstract] OR "clinical decision support"[Title/Abstract] OR "decision support system"[Title/Abstract]) AND (perioperative[Title/Abstract] OR anesthesia[Title/Abstract] OR anaesthesia[Title/Abstract] OR "intensive care"[Title/Abstract] OR ICU[Title/Abstract])) AND ("2016/01/01"[Date - Publication] : "2026/08/31"[Date - Publication]) AND english[Language]
4	**AI / Machine Learning in Anesthesia and Critical Care**	(("artificial intelligence"[Title/Abstract] OR "machine learning"[Title/Abstract] OR "deep learning"[Title/Abstract]) AND (anesthesia[Title/Abstract] OR anaesthesia[Title/Abstract] OR perioperative[Title/Abstract] OR "critical care"[Title/Abstract] OR "intensive care"[Title/Abstract])) AND ("2016/01/01"[Date - Publication] : "2026/08/31"[Date - Publication]) AND english[Language]
5	**Intraoperative Hypotension Prediction**	(("intraoperative hypotension"[Title/Abstract] OR "hypotension prediction"[Title/Abstract] OR "hypotension prediction index"[Title/Abstract]) AND ("artificial intelligence"[Title/Abstract] OR "machine learning"[Title/Abstract] OR predict*[Title/Abstract] OR "early warning"[Title/Abstract])) AND ("2016/01/01"[Date - Publication] : "2026/08/31"[Date - Publication]) AND english[Language]

6	**CRRT / Pharmacokinetics / Drug Dosing**	(("continuous renal replacement therapy"[Title/Abstract] OR CRRT[Title/Abstract]) AND ("drug dosing"[Title/Abstract] OR dosing[Title/Abstract] OR pharmacokinetic*[Title/Abstract] OR "drug clearance"[Title/Abstract] OR "therapeutic drug monitoring"[Title/Abstract])) AND ("2016/01/01"[Date - Publication] : "2026/08/31"[Date - Publication]) AND english[Language]
7	**Model-Informed Precision Dosing / TDM / Bayesian Dosing**	(("model-informed precision dosing"[Title/Abstract] OR "model informed precision dosing"[Title/Abstract] OR "precision dosing"[Title/Abstract] OR "Bayesian dosing"[Title/Abstract] OR "Bayesian dose"[Title/Abstract] OR "therapeutic drug monitoring"[Title/Abstract]) AND ("critical care"[Title/Abstract] OR "critically ill"[Title/Abstract] OR "intensive care"[Title/Abstract] OR ICU[Title/Abstract])) AND ("2016/01/01"[Date - Publication] : "2026/08/31"[Date - Publication]) AND english[Language]
8	**Digital Twin in Critical Care / Perioperative Care**	(("digital twin"[Title/Abstract] OR "digital twins"[Title/Abstract]) AND ("critical care"[Title/Abstract] OR "intensive care"[Title/Abstract] OR ICU[Title/Abstract] OR perioperative[Title/Abstract] OR anesthesia[Title/Abstract] OR anaesthesia[Title/Abstract])) AND ("2016/01/01"[Date - Publication] : "2026/08/31"[Date - Publication]) AND english[Language]
9	**LLM / Generative AI / Agentic AI**	(("large language model"[Title/Abstract] OR "large language models"[Title/Abstract] OR "generative artificial intelligence"[Title/Abstract] OR "generative AI"[Title/Abstract] OR "agentic AI"[Title/Abstract] OR "agentic artificial intelligence"[Title/Abstract]) AND ("critical care"[Title/Abstract] OR "intensive care"[Title/Abstract] OR ICU[Title/Abstract] OR perioperative[Title/Abstract] OR anesthesia[Title/Abstract] OR anaesthesia[Title/Abstract])) AND ("2016/01/01"[Date - Publication] : "2026/08/31"[Date - Publication]) AND english[Language]
10	**HL7 FHIR / SNOMED CT / LOINC**	(("HL7 FHIR"[Title/Abstract] OR FHIR[Title/Abstract] OR "SNOMED CT"[Title/Abstract] OR LOINC[Title/Abstract]) AND (perioperative[Title/Abstract] OR anesthesia[Title/Abstract] OR anaesthesia[Title/Abstract] OR "critical care"[Title/Abstract] OR "intensive care"[Title/Abstract] OR ICU[Title/Abstract])) AND ("2016/01/01"[Date - Publication] : "2026/08/31"[Date - Publication]) AND english[Language]

Note: PubMed was the primary bibliographic database used for the structured final literature update. Google Scholar and reference-list screening were used as complementary sources during the initial iterative literature identification; the complete original query history and source-specific retrieval counts were not prospectively retained. Earlier seminal publications were considered separately when necessary to support foundational concepts, as described in the Methods.
